# Using Protein Dimers to Maximize the Protein Hybridization Efficiency with Multisite DNA Origami Scaffolds

**DOI:** 10.1371/journal.pone.0137125

**Published:** 2015-09-08

**Authors:** Vikash Verma, Leena Mallik, Rizal F. Hariadi, Sivaraj Sivaramakrishnan, Georgios Skiniotis, Ajit P. Joglekar

**Affiliations:** 1 Cell and Developmental Biology, University of Michigan Medical School, Ann Arbor, Michigan, United States of America; 2 Life Sciences Institute and Department of Biological Chemistry, University of Michigan Medical School, Michigan, United States of America; Florida State University, UNITED STATES

## Abstract

DNA origami provides a versatile platform for conducting ‘architecture-function’ analysis to determine how the nanoscale organization of multiple copies of a protein component within a multi-protein machine affects its overall function. Such analysis requires that the copy number of protein molecules bound to the origami scaffold exactly matches the desired number, and that it is uniform over an entire scaffold population. This requirement is challenging to satisfy for origami scaffolds with many protein hybridization sites, because it requires the successful completion of multiple, independent hybridization reactions. Here, we show that a cleavable dimerization domain on the hybridizing protein can be used to multiplex hybridization reactions on an origami scaffold. This strategy yields nearly 100% hybridization efficiency on a 6-site scaffold even when using low protein concentration and short incubation time. It can also be developed further to enable reliable patterning of a large number of molecules on DNA origami for architecture-function analysis.

## Introduction

**Fig 1:**
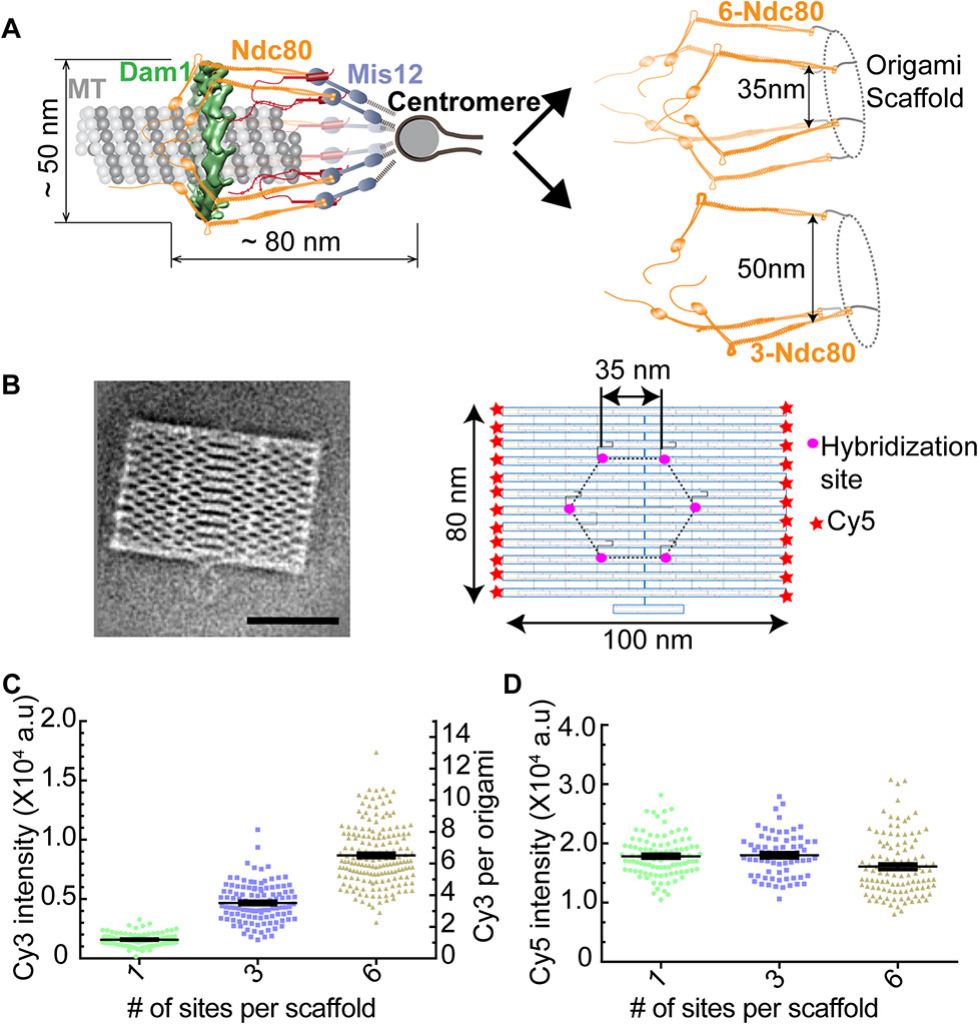
Using DNA origami scaffolds for the architecture-function analysis of the yeast kinetochore. (A) *In vivo* architecture of the yeast kinetochore-microtubule attachment (left), and schematic of the desired in vitro reconstitution and alteration of this architecture (right). (B) 2-D class average of the EM image (left, scale bar- 50 nm), and salient features of origami (right). (C) Scatter plot of intensities for Single-particle TIRF measurements (n>101, horizontal line represents the mean and the vertical line s.e.m in this and all the scatter plots that follow). The corresponding number of Cy3 molecules displayed on the right y-axis. (D) Unimodal Cy5 intensity distribution reveals that the analyzed scaffolds were monomeric.

The ability to pattern protein molecules in a precisely defined nanoscale geometry is critical for the *in vitro* reconstitution of multi-protein machines. Such reconstitution enables a systematic investigation of the functional significance of protein architecture. Specifically, how (a) the copy number of the protein, (b) the separation of one protein molecule from its nearest neighbor, and (c) the nanoscale distribution of protein molecules about the average position, affect the mechanism of protein function and its regulation. The necessity of such analysis is exemplified by the kinetochore (Fig 1A, left), which requires a precise organization of its component proteins to drive chromosome movement and segregation during cell division [1,2]. To understand how the nanoscale architecture of kinetochore components shapes the mechanisms underlying kinetochore function and regulation, an *in vitro* method that can reproduce and alter the architecture of the kinetochore is necessary.

DNA origami provides a versatile scaffold for patterning protein molecules with exquisite positional control [3–7]. DNA origami scaffolds are constructed by folding a long single stranded DNA molecule into a well-defined 2-D or 3-D shape with the help of many short, single-stranded oligonucleotides known as staple strands [3,8,9]. Some of the staple strands, selected because of their location in the final shape of the folded origami scaffold, can be extended in length and functionalized, such that they provide a site for either base-pairing with commentary single-stranded oligonucleotides or for directly with protein molecules. In this manner, functionalized origami scaffolds provide a well-defined coordinate system for precisely patterning proteins and conducting architecture-function analysis.

When a large number of protein molecules are to be patterned on an origami scaffold, highly efficient and uniform hybridization can prove to be challenging. Even when the probability of single site hybridization is 90%, the average probability of hybridizing protein with all the sites on every scaffold (which is termed here as hybridization efficiency) can be significantly lower (as low as 53% for a 6-site scaffold). Moreover, the protein-bound scaffold population is also heterogeneous. It contains scaffolds with lower than expected numbers of hybridized proteins that are randomly distributed amongst the designed positions on the scaffold [5]. This heterogeneity in number and position can complicate architecture-function analysis. To overcome this challenge, one must use hybridization conditions involving a combination of high protein concentrations, which can be difficult to obtain for many proteins, and long incubation periods, which can reduce protein activity. Therefore, we tested whether artificial dimerization of the protein to be hybridized can improve the population-wide hybridization efficiency with multi-site origami scaffolds even when using modest protein concentration and short incubation periods.

## Materials and Methods

### Cloning

The Sequoia complex, an engineered version of the Ndc80 complex, was a generous gift from Dr. Andrea Musacchio. It consists of two subunits created by fusing Nuf2 (1–364) with Spc24 (84–213), and Ndc80 (1–584) with Spc25 (57–222). The ORFs of these engineered subunits were sub-cloned into the pGEX-6P expression vector. The Nuf2-Spc24 fusion protein contains the Glutathione S-Transferase (GST) tag at its N-terminus to enable protein purification. We introduced the SNAPf domain (NEB) at the C-terminus of Ndc80-Spc25.

### Sequoia complex purification

The Sequoia complex was expressed in BL21-Rosetta 2 (DE3) *E*.*coli* cells (Novagen) using 0.3 mM IPTG for 16 hours at 18°C. Cells were then harvested, and resuspended in the lysis buffer (50 mM HEPES-KOH (pH 7.5), 300 mM NaCl, 1mM EDTA, 0.1% NP-40 (Sigma) 10% glycerol, 1 mM DTT, 1 mM PMSF) supplemented with complete EDTA-free protease inhibitor mix (Roche). Cells were lysed by sonication (3X for 3 minutes). Cleared supernatant was incubated with GST agarose beads (Pierce) for 2 h at 4°C. Beads were washed with lysis buffer containing 0.5% NP-40, and protein was eluted with lysis buffer containing 50 mM reduced glutathione. Subsequently, the protein was loaded onto a 16/60 Superdex 200 size exclusion column (GE) equilibrated with gel filtration buffer (20 mM HEPES-KOH (pH 7.5), 150 mM NaCl, 10% glycerol, 1 mM DTT). Fractions were analyzed by SDS-PAGE and Coomassie staining (panel A in S3 Fig), and peak fractions were aliquoted and stored at −80°C. The GST tag was removed from Sequoia complex by incubating the protein with PreScission Protease (GE) for 12 hours at 4°C when needed (panel B in S3 Fig).

### Preparation of benzyl-guanine (BG) conjugated oligonucleotides

5’-amino modified oligonucleotides with a Cy3 fluorophore attached at the 3’ end (IDT DNA) were covalently linked to benzyl-guanine NHS-ester (BG-GLA-NHS (NEB)) by incubating 0.84 mM oligo and 11.5 mM BG-GLA-NHS in 0.1M NaBO3 (pH-8.5) for 2.5 hours at 25°C with shaking. To remove excess BG-NHS, samples were passed through Micro Bio-Spin 6 columns (Bio-Rad) at least three times. Final concentration of BG-oligonucleotides was determined by measuring UV absorbance at 260 nm. Aliquots of BG-oligonucleotides were stored at -20°C until further use.

### Conjugation and Purification of Sequoia-DNA complex

Purified Sequoia-SNAPf complex was incubated with 18 μM of BG-oligonucleotide for 16 hours at 4°C. To remove unconjugated BG-oligonucleotides, Sequoia-BG-oligo ensemble was bound to glutathione agarose beads for 2 hours at 4°C. These beads were then washed three times with wash buffer (20mM HEPES-7.5, 150mM NaCl, 5% Glycerol and 1mM DTT). Sequoia-BG-oligo ensemble was eluted with GST elution buffer (50 mM reduced Glutathione, 20 mM HEPES, pH 7.5, 150 mM NaCl, 5% Glycerol and 1 mM DTT). Labeling was confirmed by running the Sequoia-oligo complex on a SDS-agarose gel followed by measurement of Cy3 fluorescence using a Typhoon FLA 7000 laser scanner (GE).

### Assembly of 2D-DNA origami

M13mp18 single-stranded DNA (NEB) was used as scaffold strand. All DNA oligonucleotides (staple strands) were purchased from IDT. The assembly of DNA origami was achieved by mixing 100 nM of scaffold strand with a combination of staple strands (1μM each) in a TAE buffer containing 12.5 mM of MgCl_2_. Subsequently, the mixture was subjected to gradient thermal annealing (from 90°C to 30°C in 2 hours) using a thermal cycler (Bio-Rad). The integrity of assembly was verified by running a SDS agarose gel.

### Hybridization of the BG-oligo-Sequoia complex to the origami scaffold

Sequoia-BG oligo complex (~100 nM) was mixed with 1 μL of (~20nM) DNA origami scaffold supplemented with 1 mg/ml BSA in TAE. The mixture was incubated at 35°C for 20 min with shaking to achieve maximum hybridization. After incubation, the mixture was cooled down to room temperature in ~ 10 min, and immediately used either for the gel shift assay or single molecule TIRF imaging.

### Removal of unbound Sequoia

Unbound Sequoia complex was purified as described before [6]. Briefly, Sequoia-origami ensemble was first hybridized to biotin-conjugated ss-DNA, and then immobilized on streptavidin-coated magnetic beads (NEB) for 30 minutes at room temperature. These beads were washed 3 times with wash buffer (20mM HEPES-7.5, 150mM NaCl, 5% Glycerol and 1mM DTT) to remove excess Sequoia complex that was not hybridized to the scaffold. Sequoia-origami ensemble was released by incubating the beads with a competitive oligonucleotide strand (200 nM).

### Electrophoretic mobility assay

The Sequoia-origami ensemble was incubated with 2X Laemmli buffer containing 12.5mM MgCl_2_ for 2 minutes and the sample was run on SDS-agarose gel (0.7% agarose, 0.1% SDS and 12.5 mM MgCl_2_) for 4 hours at 75 volts in TAE buffer supplemented with 0.1% SDS and 12.5mM MgCl_2_. The gel was then scanned using the Cy3 and Cy5 channels on the Typhoon FLA 7000 laser scanner (GE). Band intensities were quantified using the Image J gel quantification plug-in.

### TIRF microscopy

All images were acquired on a Nikon Ti-E microscope equipped with a 100X 1.4 NA CFI-Apo oil immersion objective, an EMCCD camera (iXon^+^ DU 897; Andor), an 3 line (488, 561 and 640nm) monolithic laser combiner with AOTF laser system (Agilent) and Nikon NIS-Elements software. Images of Cy5 tethered origami and Cy3 conjugated Sequoia complex were acquired at the following settings: 600 frames @ 50ms exposure time, conversion gain-1X, EM multiplier gain setting 288, 561 laser power-40% and 640 laser power-20%.

### Sample preparation for TIRF imaging

Flow cells were created on a clean glass slide (Thermo Scientific) by sticking two stripes of double-sided tape and plasma cleaned microscopic coverslips (Thermo Scientific). Flow cells were first incubated with 30 μl of 1 mg/mL biotinylated BSA (Sigma) for 10 minutes in a humidified chamber, followed by another incubation of 0.5 mg/mL Neutravidin (Invitrogen), and finally with 1 mg/mL BSA to block non-coated sites. These blocked flow cells were then incubated with biotinylated DNA origami tethered with Sequoia complex for 25 minutes, and then washed extensively with wash buffer (20mM HEPES-7.5, 150mM Nacl, 5% Glycerol, 1mg/mL BSA). Subsequently, chambers were sealed and samples were immediately imaged using TIRF.

### Photobleaching experiment

Origami ensembles containing Cy3 conjugated sequoia complexes were imaged using 561nm laser and an EMCCD camera (iXon^+^ DU 897; Andor) in continuous mode with 50ms exposure time for 600 frames. During this time period most of the Cy3 molecules photobleached to background levels. Photobleaching traces were generated using an automated script that calculates the estimated local background fluorescence for each frame and subtracts it from the fluorescence signal. To reveal photobleaching steps, the raw signal was filtered using the Chung-Kennedy method with a window width of 10.

### Image analysis

All images were analyzed using a custom-written graphical user interface in MATLAB based on the method described previously [10]. Briefly, a 6x6 pixel box centered on the brightest four pixels in a user-selected spot in the Cy5 channel was used to measure the fluorescence signal from a single particle. The local background was estimated from the average of the peripheral pixels in a 10x10 box placed concentrically with the signal box, and then subtracted from each signal pixel. The average signal from the first 10 frames for each particle was used for further analysis.

### Electron Microscopy

3 μl of sample was applied to a freshly glow-discharged carbon-coated grid. Sample was incubated for 1 min at room temperature; excess sample was removed by blotting off with the filter paper and stained with 0.75% (w/v) of uranyl formate (UF). Grids were imaged using Tecnai T12 transmission electron microscope operated at a voltage of 120 kV. Images were recorded at a magnification of 50,000X and a defocus value of-1.0 μm on a Gatan US4000 CCD camera. All images were binned over 2 × 2 pixels to obtain a pixel size of 4.16 Å on specimen level.

For DNA origami, 1,421 particles were picked manually from the binned micrographs by using boxer of the EMAN 1.9 software suite [11]. Particles were then subjected to reference-free 2D classification using EMAN 1.9 image processing suite [11] and classified into 10 classes.

### Statistical Analysis

All statistics were calculated using Prism data analysis software (GraphPad Prism). Log-Gaussian fit was applied to the frequency distribution of oligo conjugated origami scaffolds using Prism [12].

## Results and Discussion

To conduct architecture-function analysis of the yeast kinetochore, we selected a previously described 2-D DNA origami scaffold (average EM of negatively stained origami shown in Fig 1B and panel C in S3 Fig) that provides up to six sites for protein hybridization [3,4,6]. The number as well as spacing of hybridization sites on this scaffold can be easily adapted to meet experimental needs. Therefore, it is well-suited for the architecture-function analysis for the yeast kinetochore containing 6–8 copies of its component protein complexes (Fig 1A, right). Our initial goal was to organize the Ndc80 complex, a key component of the yeast kinetochore, into a kinetochore-like assembly using DNA origami [13].

We first tested whether all six functionalized staple strands on the selected origami scaffold can base-pair with complementary oligos, such that 100% hybridization efficiency can be realized for the entire scaffold population. We incubated scaffolds with 1, 3, and 6 hybridization sites with complementary oligonucleotides conjugated with the Cy3 fluorophore. Using Total Internal Reflection Fluorescence (TIRF) microscopy and single particle analysis, we quantified the cumulative Cy3 fluorescence from individual origami particles in each case (Fig 1C). We deduced the number of hybridized oligos in each case by comparing the cumulative fluorescence from individual particles with that of a single Cy3-conjugated oligonucleotide that was adhered to the coverslip determined using identical imaging conditions (panel A in S1 Fig). We found that the average number of oligos hybridized to each scaffold matched the expected number of hybridization sites (1.1 ± 0.4, 3.4 ± 0.1, and 6.4 ± 0.1, mean ± s.e.m., respectively for the 1, 3 and 6-site scaffolds, Fig 1C, S1 Table). We also verified the number of bound oligos by observing stepwise photobleaching (panel B in S1 Fig). Finally, we ensured that only monomeric scaffolds were selected in this analysis by using the fluorescence of Cy5 molecules as the reference (Fig 1D and panel C in S2 Fig). These data show that the origami scaffold provides six sites that can be occupied with 100% hybridization efficiency.

The high hybridization efficiency (~100%) in the above experiment was facilitated by the high oligo concentration used (18 μM). Such high concentration is not easily obtainable for proteins. Consequently, the hybridization efficiency with the same origami scaffold can be significantly lower for proteins. We tested this prediction for the Sequoia complex constructed by the Musacchio lab [14], which is an engineered version of a critical component of the kinetochore, the Ndc80 complex (Fig 1B). It was constructed by fusing the α-helices of Ndc80 complex subunits together to reduce the number of subunits from 4 to 2. In addition, the Glutathione S-Transferase (GST) domain is fused to the N-terminus of Nuf2-Spc24 fusion to enable its purification. Importantly, the Sequoia complex preserves the overall domain organization of the Ndc80 complex, and also displays a comparable length (~ 47 vs 57 nm, panel D in S3 Fig). We fused the SNAPf domain at the C-terminus of Spc25 to allow covalent linkage to oligos functionalized with benzyl-guanine (BG) [15]. This position of the SNAPf domain ensures that the Sequoia complex can be hybridized with the DNA origami in a conformation that mimics the conformation and orientation of the Ndc80 complex in the yeast kinetochore (Fig 1A).

Even with its simplified design, protein yield for the Sequoia complex was significantly lower than oligo concentration used above (~ 500 nM). Due to the strong dimerization of GST domains, we expected that the purified Sequoia complex would be dimeric. This was confirmed by gel filtration experiments wherein the purified Sequoia complex (expected molecular weight = 350 kDa) eluted as a single peak immediately after Ferritin (443 kDa) (panel A in S3 Fig). The enhanced mobility of the complex is due to its highly elongated shape. Sequoia monomers are easily obtained by proteolytically removing the dimerizing GST domain from the Sequoia complex (panel B in S3 Fig).

**Fig 2:**
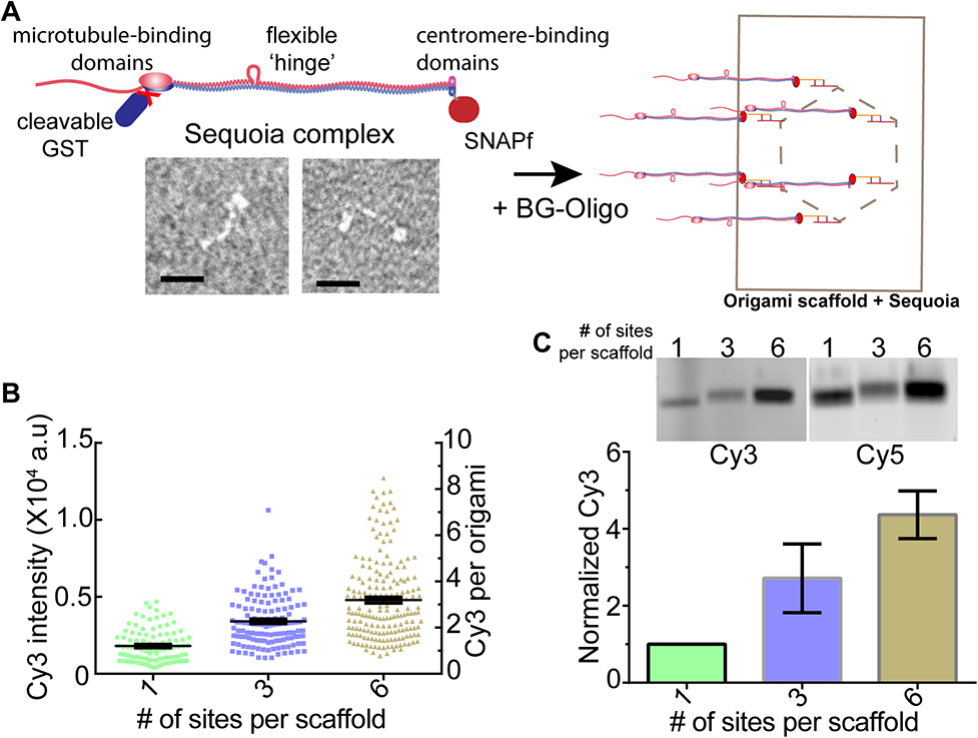
Hybridization of the Sequoia complex with the DNA origami scaffold. (A) Schematic (top left) and negative stained EM of the Sequoia complex (bottom left, scale bar-20nm), and the proposed reconstitution of the in vivo Ndc80 complex architecture using the Sequoia complex and the 6-site origami scaffold (right). (B) Fluorescence intensity and the deduced number of Cy3 molecules per scaffold (n ≥ 99). (C) Mobility (top) and intensity analysis (bottom) of the scaffolds hybridized with the Sequoia complex (n = 3).

To assess the hybridization efficiency for the Sequoia complex with the 1, 3, and 6-site scaffolds, we first cleaved the GST tag, conjugated the resulting monomeric Sequoia with Cy3-oligos, and then hybridized it with the three scaffolds. Using fluorescence quantitation, we found that the 1- and 3-site scaffolds bound 1.3 ± 0.1 and 2.5 ± 0.1 Cy3 molecules respectively (Fig 2B). Thus, the hybridization efficiency was high in these two cases. However, the 6-site scaffold bound only 3.5 ± 0.1 Cy3 molecules, corresponding to the hybridization efficiency of only 60%. To ensure that single particle analysis represented the entire population, we examined the mobility and fluorescence of the hybridized scaffolds on SDS-agarose gels (Fig 2C). In agreement with the microscopy data, the mobility of the 6-site scaffold did not appear to be significantly different from that of the 3-site scaffold (panel D in S4 Fig). Quantitation of the Cy3 and Cy5 fluorescence from the gel bands confirmed that the maximal occupancy of the 6-site scaffold was ~ 60% (Fig 2C and panel B in S4 Fig). Thus the majority of the 6-site scaffolds in these experiments carry 3–4 Sequoia molecules. However, this population is also expected to be heterogeneous, and contain scaffolds with 1 or 2 molecules. However, the relatively small shift in the mobility of the scaffold due to each Sequoia complex makes it difficult to examine the heterogeneity in the scaffold population based on mobility alone.

These data suggest that the concentration of the reconstituted Sequoia may not be sufficiently high to obtain efficiency 100% hybridization efficiency for the 6-site origami scaffold. To test if the low efficiency observed is mainly due to a lower than desirable protein concentration, we quantified the hybridization efficiency for fluorescently labeled DNA oligos with the 1, 3 and 6-site scaffolds when the oligo concentration was comparable to the protein concentration used above (100 nM). The hybridization efficiency in this case was comparable to that of the monomeric Sequoia complex (1.1 ± 0.1, 1.7 ± 0.1 and 3.1 ± 0.1, S2 Fig). Thus, the low concentration of the Sequoia complex was primarily responsible for the low hybridization efficiency observed in the case of the 6-site scaffold.

Although the above experiments yielded sub-optimal hybridization efficiency, they nonetheless provided a key insight. Concentration of the Sequoia complex used above was sufficiently high to yield 100% hybridization efficiency for the 1-site scaffold, and 80–90% for the 3-site scaffold under the assay conditions used. Therefore, we reasoned that the efficiency for the 6-site scaffold may be low because the low rate of the hybridization of the protein with the scaffold limits the number of hybridization events that can take place over the incubation period used. It should be noted that the hybridization of the 28-nucleotide long oligos with the scaffold is nearly irreversible under the experimental conditions (*ΔG* = 32.3 kcal/mole). Hence, the hybridization efficiency for a given protein concentration can be improved by: (a) using longer incubation period, and (b) using dimeric Sequoia. If the initial encounter is indeed the rate limiting step, then the use of dimeric Sequoia can also improve the hybridization efficiency. This will ensure that the hybridization of one of the protein molecules with the dimer will facilitate the hybridization of the second molecule. We expected that the second hybridization event would not suffer from steric limitations, because the extensive length of the Sequoia complex (~ 50 nm) and the position of the dimerization domain relative to the SNAPf domain will make adjacent hybridization sites on the scaffold easily accessible.

**Fig 3:**
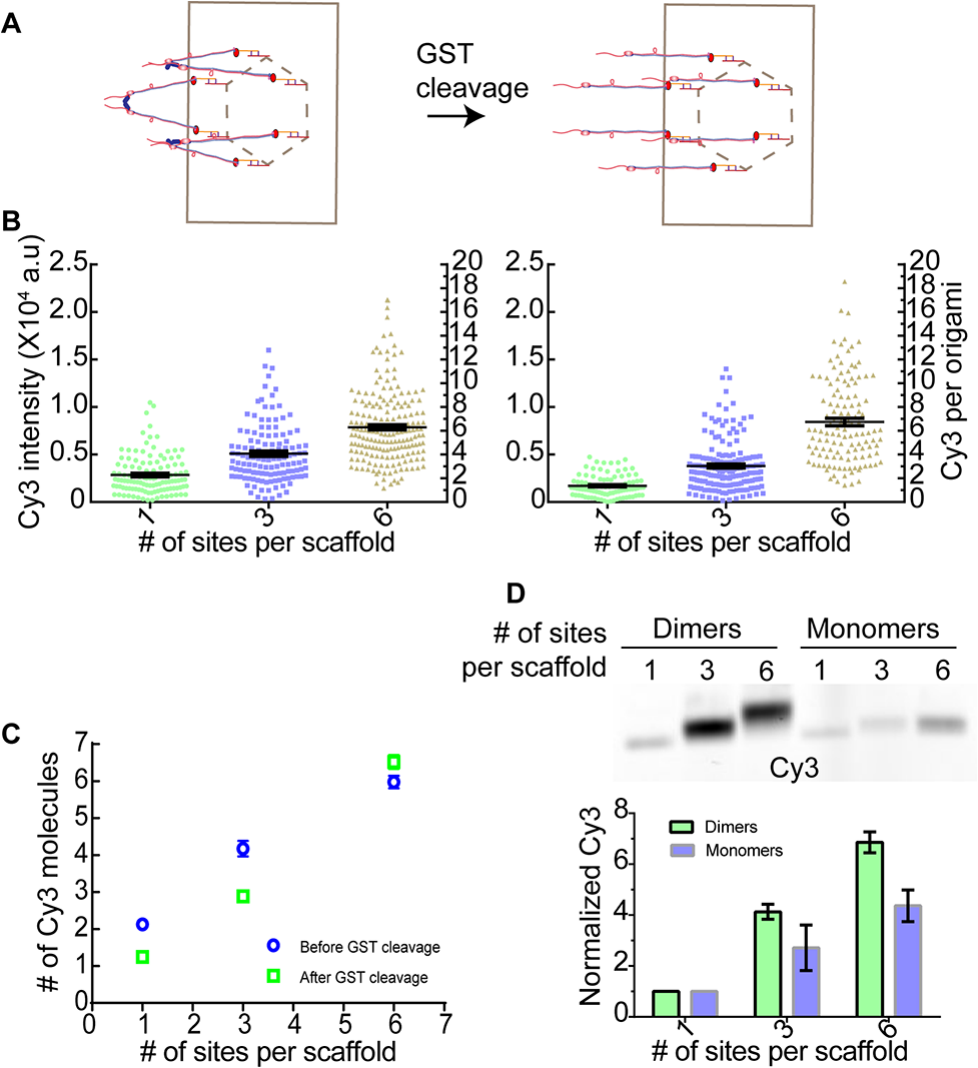
Exploiting GST dimerization for maximizing hybridization efficiency. (A) Schematic of dimeric (left) and monomeric (right) Sequoia hybridized to origami scaffold. (B) Single particle Cy3 fluorescence and the number of molecules for dimeric Sequoia hybridization before (left, n≥117) and after GST removal (right, n≥92). (C) The number of Sequoia molecules per scaffold from B (mean ± s.e.m). (D) Mobility of scaffolds conjugated with dimeric and monomeric Sequoia molecules (top) and Cy3 intensity quantitation (bottom, n = 3).

To test the effectiveness of this strategy, we conjugated dimeric Sequoia complex to the 1, 3, and 6-site scaffold. To obtain a functional, patterned assembly of proteins, the GST domains can be cleaved after conjugation (Fig 3A). In this experiment, we expected that hybridization of dimeric Sequoia with the 1-site scaffold should bind two Sequoia molecules rather than one. The exact number of molecules bound to the 3- and 6-site scaffolds depends on the relative magnitudes of two quantities: the rate of successful hybridization of a free Sequoia dimer with a free scaffold, and the rate at which a Sequoia molecule that is already tethered to the scaffold (via a successful hybridization of its partner to the scaffold) hybridizes with one of the remaining hybridization sites on that scaffold. If the former rate is much higher that the latter, then we expected that each hybridization site on the 3- and 6-site scaffolds would hybridize with a Sequoia dimer. Therefore, the total number of Sequoia molecules associated with each scaffold would be 6 and 12 respectively. On the other hand, if the second rate is significantly higher, the successful hybridization of one of the Sequoia molecules in the dimer will couple the hybridization of the second molecule. Therefore, in this case, the 3 and 6-site scaffolds will bind 4 and 6 Sequoia molecules respectively. It should be noted that the tethering of the Sequoia molecule to the scaffold will increase its effective concentration for the hybridization sites on the same scaffold by several orders of magnitude. Therefore, given the low protein concentrations used, we expected that the enhancement in binding would be on the lower side of the possible range predicted by the two scenarios discussed above.

As expected, single particle fluorescence quantitation revealed that the 1-site scaffold was conjugated to 2.1 ± 0.1 Cy3 molecules (Fig 3B). The 3 and 6-site scaffolds were now conjugated to 3.8 ± 0.2 and 5.8 ± 0.2 Cy3 molecules respectively. Thus, most of the hybridization events were indeed coupled on these scaffolds: hybridization of one Sequoia molecule in the dimer greatly facilitated the hybridization of the second Sequoia molecule. The hybridization efficiency was also significantly improved, especially for the 6-site scaffold. These findings were further bolstered by the retarded mobility of the hybridized scaffolds on SDS agarose gels and fluorescence quantitation of the gel. These measurements confirmed that the improved hybridization efficiency was obtained for the entire scaffold population (Fig 3D).

In addition to high hybridization efficiency, removal of the dimerizing GST tag from the hybridized Sequoia molecules is critical for releasing any unhybridized protein and obtaining a biologically active assembly. To test this, we incubated the scaffolds hybridized with dimeric Sequoia with a site-specific protease (panel A in S4 Fig), and quantified the Cy3 fluorescence per scaffold before and after GST removal (Fig 3B and 3C, S1 Table). We found that the number of Cy3 molecules per scaffold decreased from 2.1 ± 0.1 to 1.2 ± 0.1 for the 1-site scaffold, and from 3.8 ± 0.2 to 2.8 ± 0.15 for the 3-site scaffold. As expected, this number did not change significantly for the 6-site scaffold (5.8 ± 0.2 versus 6.2 ± 0.3), (Fig 3C). These data demonstrate that the removal of the GST tag was successful, and a patterned assembly of the Sequoia complex was obtained.

## Conclusion

Our assay provides a systematic method for assessing the protein hybridization efficiency of multisite origami scaffolds, and improving it with the help of a cleavable dimerization domain. A potential issue with use of dimeric or multimeric proteins is that, if the protein concentration is sufficiently low, proteins in the dimer/multimer could hybridize with more than one origami scaffold and lead to aggregation. Even in such cases, post-processing of the hybridized origami scaffolds (e.g. density gradient centrifugation or Gel filtration) can be used to purify the desired assemblies of proteins [16,17]. Our strategy will be especially useful for the high efficiency hybridization of proteins to scaffolds with a large number of hybridization sites, for hybridizing proteins that cannot be obtained at high concentration, or when incubation time must be minimized. It can also be optimized further to pattern scaffolds with larger numbers of proteins. This work also forms the foundation for our systematic, in vitro architecture-function analysis of the yeast kinetochore.

## Supporting Information

S1 Fig Single molecule characterization of Cy3-oligos base-paired with the origami scaffold. (A) Top: TIRF micrographs of Cy3 (oligo) and Cy5 (origami scaffold) channels for the 1- and 6-site scaffolds conjugated with Cy3-oligos. Histograms of single particle Cy3 intensity for 1-site (left) and 6-site (right) DNA origami scaffold. Solid line displays a log-Gaussian fit (peak for 1-site = 1583±53, *R* ^*2*^ = 0.94, and 8680±195, *R* ^*2*^ = 0.89 for the 6-site scaffold). Fluorescence from the 1-site scaffold was used as the fluorescence of single Cy3 molecules. (B) Green line: continuous photobleaching of Cy3 conjugated to oligonucleotides hybridized to 1-site (left) and 6site (right) scaffolds. Black line: data filtered using the Chung-Kennedy filter. Photobleaching events (arrows) are clearly visible in the filtered data.
(TIF)

S2 Fig Hybridization of Cy3 conjugated oligonucleotides with the DNA origami scaffold. (A) Fluorescence intensity and the deduced number of Cy3 molecules per scaffold (n ≥ 45) at 100nm oligo concentration. (B) Titration of oligo concentration (5nm, 50nm and 100nm) to quantify the hybridization efficiency with the origami scaffold. (C) Unimodal Cy5 intensity distribution of dimeric Sequoia hybridized to the origami scaffold.
(TIF)

S3 Fig Purification and EM characterization of the Sequoia complex. (A) GST purified Sequoia complex was applied to the Superdex-200 gel filtration column and the resulting fractions were then analyzed using coomassie stained 10% SDS-PAGE. Only those fractions containing the sequoia complex are shown. Vertical arrow heads indicate the elution volumes of marker proteins (Ferritin ~ 443 kDa and β-Amylase ~ 200 kDa). (B) Coomassie stained 10% SDS-PAGE showing purified Sequoia complex (Ndc80-Spc25 and Nuf2-Spc24) before and after GST cleavage. * indicates either protein degradation or contamination. (C) Negative stained electron micrographs of the DNA origami scaffold (left) and three representative 2-D class average images (right, scale bar—50 nm, n = 1421 particles). (D) Negative stained TEM image of Sequoia complex (left), selected images of the Sequoia complex (right, scale bar—20 nm). Note that the Ndc80 subunit of the Ndc80 complex contains a flexible ‘hinge’ domain that allows the front section of the complex to bend freely through 90°. Insets show instances of molecules with bent conformation [18], (scale bar ~ 20nm).
(TIF)

S4 Fig Validation of GST removal and quantitation of Sequoia complex attached to origami scaffold. (A) Coomassie stained SDS gel assessing GST cleavage from Sequoia-origami ensemble using the PreScission Protease (GE). Note that the BSA, which was used as a crowding agent, appears as a strong band on the gel that completely masks the band corresponding to the Nuf2-Spc24 subunit (compare with after cleavage lanes in panel B in S3 Fig). (B) SDS-Agarose gels showing Cy3 (upper panel) and Cy5 (lower panel) intensities of scaffolds incubated with either dimeric or monomeric Sequoia complex as indicated. Histogram shows the quantitation of Cy3 intensity with respect to Cy5 intensity (Cy3/Cy5). * The Cy5 intensity for the 3-site scaffold saturated the detector. Therefore, we estimated the scaffold concentration in this case by measuring the thickness of the band rather than intensity. (C) Frequency distribution of Cy3 fluorescence of 1 and 6-site Sequoia-origami ensembles (orange lines). This distribution is somewhat broader than the distributions obtained with the Cy3-oligos alone (blue lines). However, the mean fluorescence does not change significantly even if a small number of data points are discarded on the basis of the upper limit of the oligo-origami distribution (not shown). Insets show TIRF Cy3 and Cy5 micrographs for the respective samples. (D) Line scan of Cy3 gel shown in Fig 2C. There is no distinct shift of 6-site origami scaffolds hybridized with monomeric Sequoia; it resembles the distribution of the 3-site origami population. The shift is very clear in the case of dimeric Sequoia hybridization. In both cases however, the shape of the curve is asymmetric on the side with lower number of hybridized molecules. Asymmetric tails potentially represent the scaffold population with lower number of hybridized molecules.
(TIF)

S1 Table Statistical information of the data presented in the text. (DOCX)

## References

[1] Aravamudhan P, Goldfarb AA, Joglekar AP. The kinetochore encodes a mechanical switch to disrupt spindle assembly checkpoint signalling. Nat Cell Biol. 2015; 17: 868–79. 10.1038/ncb3179 26053220 PMC4630029

[2] Aravamudhan P, Felzer-Kim I, Gurunathan K, Joglekar AP. Assembling the protein architecture of the budding yeast kinetochore-microtubule attachment using FRET. Curr Biol. 2014; 24: 1437–1446. 10.1016/j.cub.2014.05.014 24930965 PMC4320969

[3] Rothemund PWK. Folding DNA to create nanoscale shapes and patterns. Nature. 2006; 440: 297–302. 10.1038/nature04586 16541064

[4] Woo S, Rothemund PWK. Programmable molecular recognition based on the geometry of DNA nanostructures. Nat Chem. 2011; 3: 620–627. 10.1038/nchem.1070 21778982

[5] Derr ND, Goodman BS, Jungmann R, Leschziner AE, Shih WM, Reck-Peterson SL. Tug-of-War in Motor Protein Ensembles Revealed with a Programmable DNA Origami Scaffold. Science. 2012; 338: 662–665. 10.1126/science.1226734 23065903 PMC3840815

[6] Hariadi RF, Cale M, Sivaramakrishnan S. Myosin lever arm directs collective motion on cellular actin network. [Internet]. Proceedings of the National Academy of Sciences of the United States of America. 2014; 111: 4091–6. 10.1073/pnas.1315923111 24591646 PMC3964131

[7] Meyer R, Faesen A, Vogel K, Jeganathan S, Musacchio A NC. DNA-Directed Assembly of Capture Tools for Constitutional Studies of Large Protein Complexes. Small. 2015; 11: 2669–74, 10.1002/smll.201403544 25649737

[8] Douglas SM, Dietz H, Liedl T, Högberg B, Graf F, Shih WM. Self-assembly of DNA into nanoscale three-dimensional shapes. Nature. 2009; 459: 414–418. 10.1038/nature08165 19458720 PMC2688462

[9] Dietz H, Douglas SM, Shih WM. Folding DNA into twisted and curved nanoscale shapes. Science. 2009; 325: 725–730. 10.1126/science.1174251 19661424 PMC2737683

[10] Joglekar AP, Bouck DC, Molk JN, Bloom KS, Salmon ED. Molecular architecture of a kinetochore-microtubule attachment site. Nat Cell Biol. 2006; 8: 581–5. 10.1038/ncb1414 16715078 PMC2867088

[11] Ludtke SJ, Baldwin PR, Chiu W. EMAN: semiautomated software for high-resolution single-particle reconstructions. J Struct Biol. 1999; 128: 82–97. 10.1006/jsbi.1999.4174 10600563

[12] Mutch SA, Fujimoto BS, Kuyper CL, Kuo JS, Bajjalieh SM, Chiu DT. Deconvolving single-molecule intensity distributions for quantitative microscopy measurements. Biophys J. 2007; 92: 2926–2943. 10.1529/biophysj.106.101428 17259276 PMC1831712

[13] Ciferri C, Pasqualato S, Screpanti E, Varetti G, Santaguida S, Dos Reis G, et al Implications for Kinetochore-Microtubule Attachment from the Structure of an Engineered Ndc80 Complex. Cell. 2008; 133: 427–439. 10.1016/j.cell.2008.03.020 18455984 PMC4754795

[14] Maure JF, Komoto S, Oku Y, Mino A, Pasqualato S, Natsume K, et al The Ndc80 loop region facilitates formation of kinetochore attachment to the dynamic microtubule plus end. Curr Biol. 2011; 21: 207–213. 10.1016/j.cub.2010.12.050 21256019 PMC3052438

[15] Keppler A, Gendreizig S, Gronemeyer T, Pick H, Vogel H, Johnsson K. A general method for the covalent labeling of fusion proteins with small molecules in vivo. Nature biotechnology. 2002; 21: 86–89 10.1038/nbt765 12469133

[16] Lin C, Perrault SD, Kwak M, Graf F, Shih WM. Purification of DNA-origami nanostructures by rate-zonal centrifugation. Nucleic Acids Res. 2013; 41 10.1093/nar/gks1070 23155067 PMC3553994

[17] Shaw A, Benson E, Högberg B. Purification of Functionalized DNA Origami Nanostructures. ACS Nano. 2015; 9 (5): 4968–4975. 10.1021/nn507035g 25965916

[18] Wang H-W, Long S, Ciferri C, Westermann S, Drubin D, Barnes G, et al Architecture and Flexibility of the Yeast Ndc80 Kinetochore Complex. J Mol Biol. 2008; 383: 894–903. 10.1016/j.jmb.2008.08.077 18793650 PMC2640231

