# Supplementary figures and images for: Using Protein Dimers to Maximize the Protein Hybridization Efficiency with Multisite DNA Origami Scaffolds
Source: PLoS One. 2015 Sep 8;10(9):e0137125. doi: 10.1371/journal.pone.0137125 (PMC4562706; doi:10.1371/journal.pone.0137125)

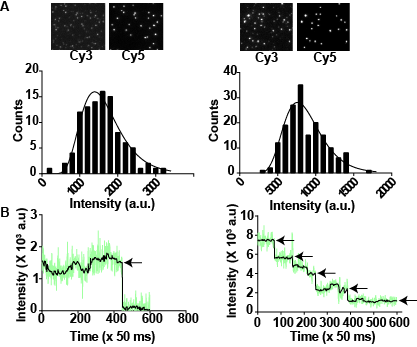

Supplement: S1 Fig — (A) Top: TIRF micrographs of Cy3 (oligo) and Cy5 (origami scaffold) channels for the 1- and 6-site scaffolds conjugated with Cy3-oligos. Histograms of single particle Cy3 intensity for 1-site (left) and 6-site (right) DNA origami scaffold. Solid line displays a log-Gaussian fit (peak for 1-site = 1583±53, R 2 = 0.94, and 8680±195, R 2 = 0.89 for the 6-site scaffold). Fluorescence from the 1-site scaffold was used as the fluorescence of single Cy3 molecules. (B) Green line: continuous photobleaching of Cy3 conjugated to oligonucleotides hybridized to 1-site (left) and 6site (right) scaffolds. Black line: data filtered using the Chung-Kennedy filter. Photobleaching events (arrows) are clearly visible in the filtered data. (TIF) [file pone.0137125.s001.tif]

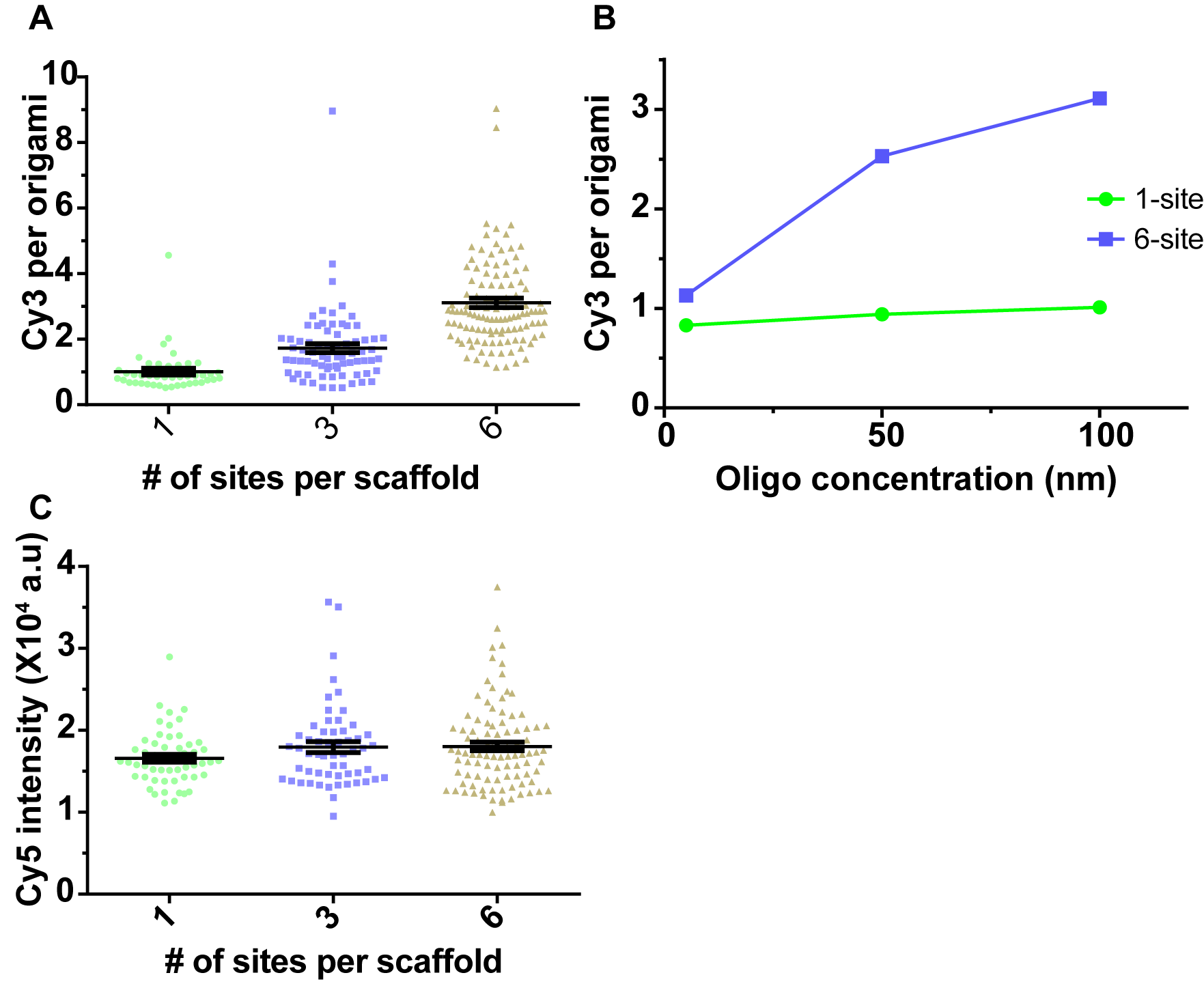

Supplement: S2 Fig — (A) Fluorescence intensity and the deduced number of Cy3 molecules per scaffold (n ≥ 45) at 100nm oligo concentration. (B) Titration of oligo concentration (5nm, 50nm and 100nm) to quantify the hybridization efficiency with the origami scaffold. (C) Unimodal Cy5 intensity distribution of dimeric Sequoia hybridized to the origami scaffold. (TIF) [file pone.0137125.s002.tif]

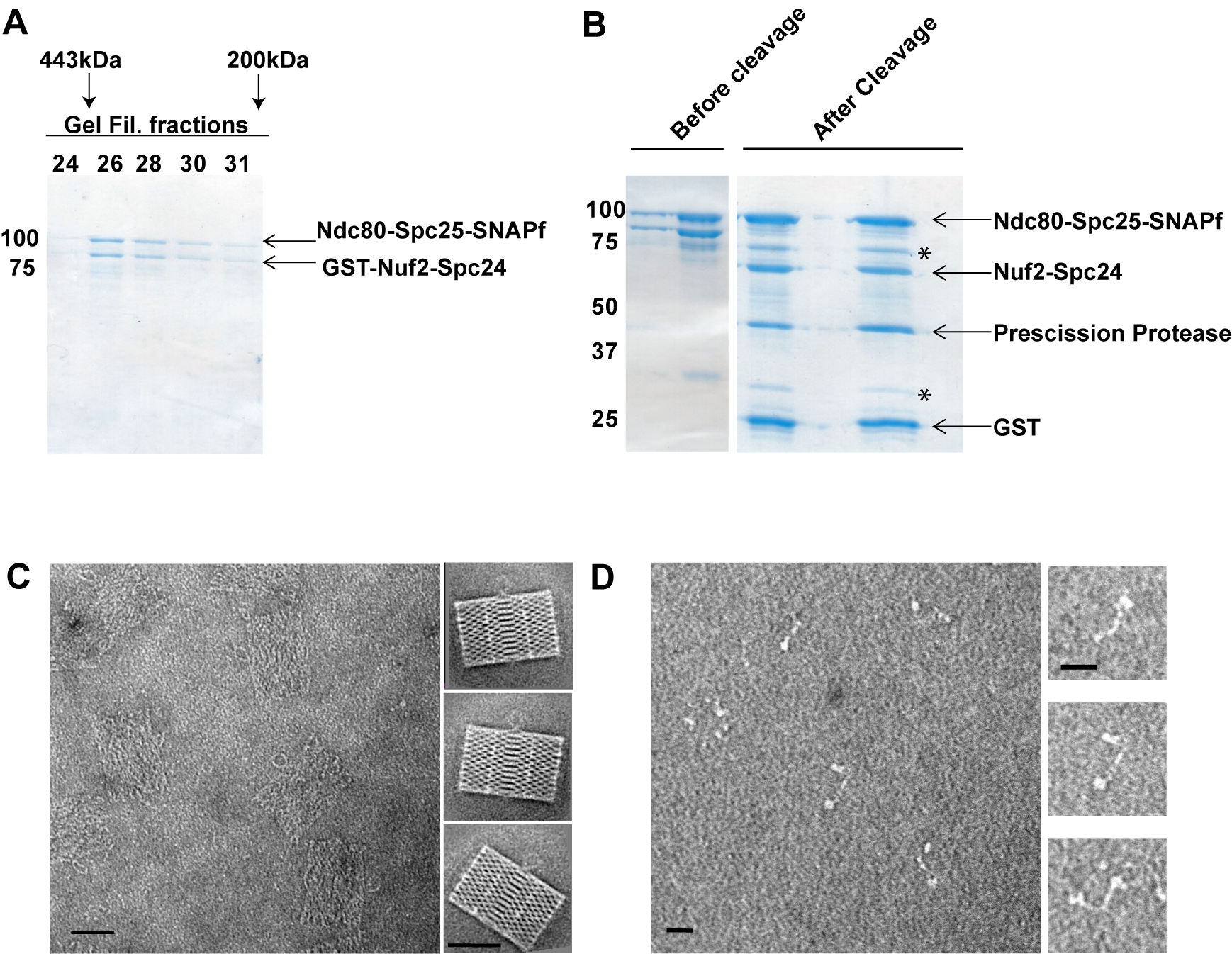

Supplement: S3 Fig — (A) GST purified Sequoia complex was applied to the Superdex-200 gel filtration column and the resulting fractions were then analyzed using coomassie stained 10% SDS-PAGE. Only those fractions containing the sequoia complex are shown. Vertical arrow heads indicate the elution volumes of marker proteins (Ferritin ~ 443 kDa and β-Amylase ~ 200 kDa). (B) Coomassie stained 10% SDS-PAGE showing purified Sequoia complex (Ndc80-Spc25 and Nuf2-Spc24) before and after GST cleavage. * indicates either protein degradation or contamination. (C) Negative stained electron micrographs of the DNA origami scaffold (left) and three representative 2-D class average images (right, scale bar—50 nm, n = 1421 particles). (D) Negative stained TEM image of Sequoia complex (left), selected images of the Sequoia complex (right, scale bar—20 nm). Note that the Ndc80 subunit of the Ndc80 complex contains a flexible ‘hinge’ domain that allows the front section of the complex to bend freely through 90°. Insets show instances of molecules with bent conformation [18], (scale bar ~ 20nm). (TIF) [file pone.0137125.s003.tif]

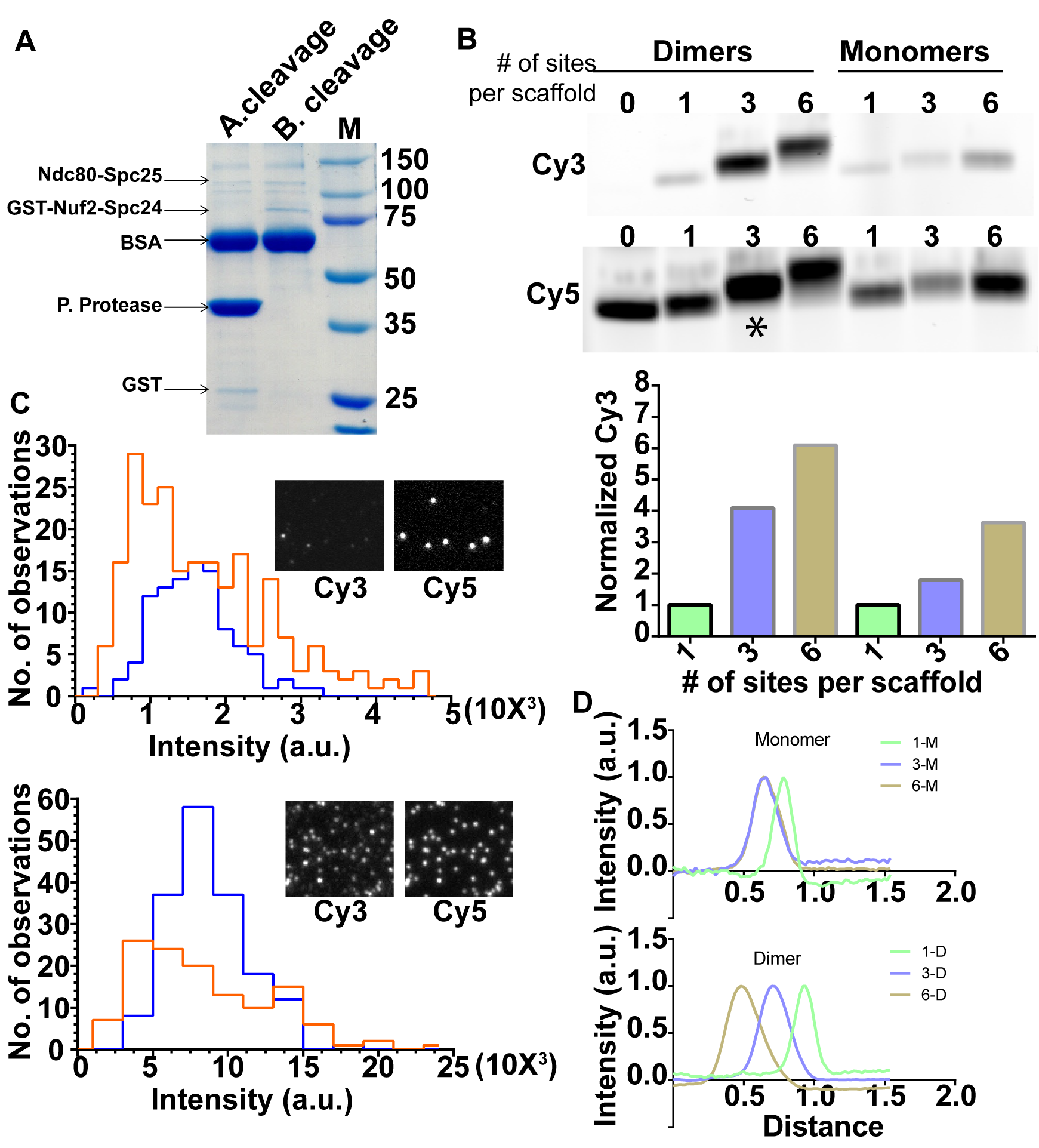

Supplement: S4 Fig — (A) Coomassie stained SDS gel assessing GST cleavage from Sequoia-origami ensemble using the PreScission Protease (GE). Note that the BSA, which was used as a crowding agent, appears as a strong band on the gel that completely masks the band corresponding to the Nuf2-Spc24 subunit (compare with after cleavage lanes in panel B in S3 Fig). (B) SDS-Agarose gels showing Cy3 (upper panel) and Cy5 (lower panel) intensities of scaffolds incubated with either dimeric or monomeric Sequoia complex as indicated. Histogram shows the quantitation of Cy3 intensity with respect to Cy5 intensity (Cy3/Cy5). * The Cy5 intensity for the 3-site scaffold saturated the detector. Therefore, we estimated the scaffold concentration in this case by measuring the thickness of the band rather than intensity. (C) Frequency distribution of Cy3 fluorescence of 1 and 6-site Sequoia-origami ensembles (orange lines). This distribution is somewhat broader than the distributions obtained with the Cy3-oligos alone (blue lines). However, the mean fluorescence does not change significantly even if a small number of data points are discarded on the basis of the upper limit of the oligo-origami distribution (not shown). Insets show TIRF Cy3 and Cy5 micrographs for the respective samples. (D) Line scan of Cy3 gel shown in Fig 2C. There is no distinct shift of 6-site origami scaffolds hybridized with monomeric Sequoia; it resembles the distribution of the 3-site origami population. The shift is very clear in the case of dimeric Sequoia hybridization. In both cases however, the shape of the curve is asymmetric on the side with lower number of hybridized molecules. Asymmetric tails potentially represent the scaffold population with lower number of hybridized molecules. (TIF) [file pone.0137125.s004.tif]
