# Supplementary material for: Using Protein Dimers to Maximize the Protein Hybridization Efficiency with Multisite DNA Origami Scaffolds
Source: PLoS One. 2015 Sep 8;10(9):e0137125. doi: 10.1371/journal.pone.0137125 (PMC4562706; doi:10.1371/journal.pone.0137125)
Supplement: S1 Table — (DOCX) [file pone.0137125.s005.docx]

**S1 Table**

| Oligonucleotides | 1-site scaffold | 3-site scaffold | 6-site scaffold |
| --- | --- | --- | --- |
| Number of observations(N) | 102 | 112 | 171 |
| Mean | 1.162 | 3.462 | 6.430 |
| Std. Error of Mean | 0.04 | 0.11 | 0.14 |

**Binding to origami after GST cleavage**

| Sequoia monomers | 1-site scaffold | 3-site scaffold | 6-site scaffold |
| --- | --- | --- | --- |
| Number of observations(N) | 99 | 130 | 189 |
| Mean | 1.320 | 2.499 | 3.510 |
| Std. Error of Mean | 0.07 | 0.10 | 0.14 |

**Binding to origami before GST cleavage**

| Sequoia dimers | 1-site scaffold | 3-site scaffold | 6-site scaffold |
| --- | --- | --- | --- |
| Number of observations(N) | 117 | 127 | 189 |
| Mean | 2.111 | 3.768 | 5.831 |
| Std. Error of Mean | 0.14 | 0.20 | 0.20 |

**After cleaving GST from hybridized Sequoia**

| Sequoia monomers | 1-site scaffold | 3-site scaffold | 6-site scaffold |
| --- | --- | --- | --- |
| Number of observations(N) | 92 | 171 | 125 |
| Mean | 1.264 | 2.798 | 6.243 |
| Std. Error of Mean | 0.08 | 0.15 | 0.29 |
